# Point of Care Ultrasound for Diagnosis and Management in Heart Failure: A Targeted Literature Review

**DOI:** 10.24908/pocus.v9i1.16795

**Published:** 2024-04-22

**Authors:** Sabina Yampolsky, Alan Kwan, Susan Cheng, Ilan Kedan

**Affiliations:** 1 Duke University Durham, NC USA; 2 Smidt Heart Institute, Cedars Sinai Hospital Los Angeles, CA USA

**Keywords:** POCUS, Heart failure, IVC, Cardiac POCUS

## Abstract

Background: Cardiac point of care ultrasound (POCUS) has shown increasing utility as a tool for diagnosing and managing heart failure (HF). Within cardiology, intravascular volume assessment leveraging visualization of the inferior vena cava (IVC) is a central aspect of care, as IVC size correlates with central venous pressure. This targeted literature review aimed to examine the existing literature assessing the use of POCUS in diagnosis and management of HF patients utilizing POCUS-based IVC measurement either alone or in combination with secondary methods. Methods: A targeted PubMed and Ovid database search up until August 28, 2023 using a keyword search was completed. Studies that did not include IVC assessment with POCUS in HF were excluded. Results: The initial search using both PubMed and Ovid resulted in 370 journal publications. After exclusion criteria were used 15 studies were included in the review. Studies were grouped into three categories: 1) how well POCUS was able to identify HF, 2) whether POCUS-based findings correlated with other measures evaluating HF and was able to predict the effect of diuretic administration, and 3) whether POCUS-based findings served as a good prognostic indicator. The 5 studies that evaluated HF identification with POCUS found that both diagnostic sensitivity and specificity may reach 90%-100% when IVC measurement was coupled with a lung ultrasound assessing the presence of B-lines or pleural effusion. Five studies assessing POCUS findings correlating with other HF measures and diuretic effect found that IVC diameter changed significantly with diuretic administration (p<0.05). All 6 studies assessing POCUS as a predictor of long-term mortality or hospital readmission found measures that achieved statistical significance with p<0.05. Conclusions: Including POCUS as standard-of-care – both as a diagnostic tool in the emergency department and a management tool in in-patient and out-patient facilities – may improve the treatment of HF.

## Introduction

Heart failure (HF) is a growing worldwide epidemic, affecting about 1-2% of the worldwide population and 2.5% of the US population 1. The mortality rate for patients diagnosed with HF is approximately 30% after 1 year following diagnosis and 45-65% after 5 years 2. As such, there is a pressing need to refine protocols for the management of HF, particularly in regard to its diagnosis and longitudinal management. 

While traditional cart-based echocardiography is well-recognized as a critical adjunct to HF management, cardiac point of care ultrasound (POCUS) is an emerging imaging modality that can provide similar qualitative and quantitative imaging of the heart, lungs, and vasculature at the bedside. Commercially available hand-carried ultrasound devices have been marketed for over 10 years 3, and POCUS with hand-carried ultrasound devices is currently applied in acute care settings for diagnosis of specific disease states like cardiac arrest, pneumothorax, pericardial effusion, and free intra-abdominal fluid in trauma or surgery patients 4, 5.

A particularly important application of POCUS is in patients with HF. Intravascular volume status can be measured by visualizing and measuring the inferior vena cava (IVC) diameter, as well as the IVC collapsibility index (IVC-CI) which is calculated using (IVC_max_ - IVC_min_)/IVC_max_. An increase in the IVC diameter as well as a reduction in the IVC-CI indicate intravascular volume overload, which is associated with cardiac dysfunction and is characteristic of HF. Furthermore, central venous pressure (CVP), which increases with impairment of right ventricular functioning, is positively correlated with IVC diameter and negatively correlated with IVC-CI 6. Possible applications of POCUS include diagnosing HF in the ED for patients presenting with dyspnea, predicting readmission and mortality outcomes for patients with HF, and serving as a more accurate management tool than brain natriuretic peptide (proBNP) levels^7^ or physical assessment of volume status. While multiple studies have evaluated the benefit of utilizing POCUS in both the diagnostic and management protocols for patients with acute decompensated heart failure (ADHF), these studies have not been systematically compared and analyzed. We provide a comprehensive review of the potential of POCUS, particularly as it is used to measure IVC, for both the hospital and outpatient settings.

## Methods

We performed a targeted literature search in PubMed and Ovid (MEDLINE and Embase) with the search term “point of care ultrasound” of “hospitalized heart failure inferior vena cava” or “point of care ultrasound” of “heart failure inferior vena cava” to identify studies that evaluated the use of POCUS, particularly with IVC measurement as a benchmark, as a diagnostic and management tool for patients hospitalized with HF. Observational cohort studies and randomized controlled studies that included adults that assessed HF, used POCUS, included a measurement of the IVC as part of their methodology, and were published in English were included in our review. The studies were grouped in terms of similar research questions and methods of analysis (Supplement). For each study, we collected data on study design, patient population, and major findings. Major findings included the correlation between POCUS measurements focusing on IVC diameter and other measurement tools, as well as the ability of POCUS to diagnose HF and predict readmission or mortality. Excluded from the review were studies that did not relate to diagnosing or managing HF, studies that did not include IVC measurement, case series, and case studies.

## Results

**Figure 1:**
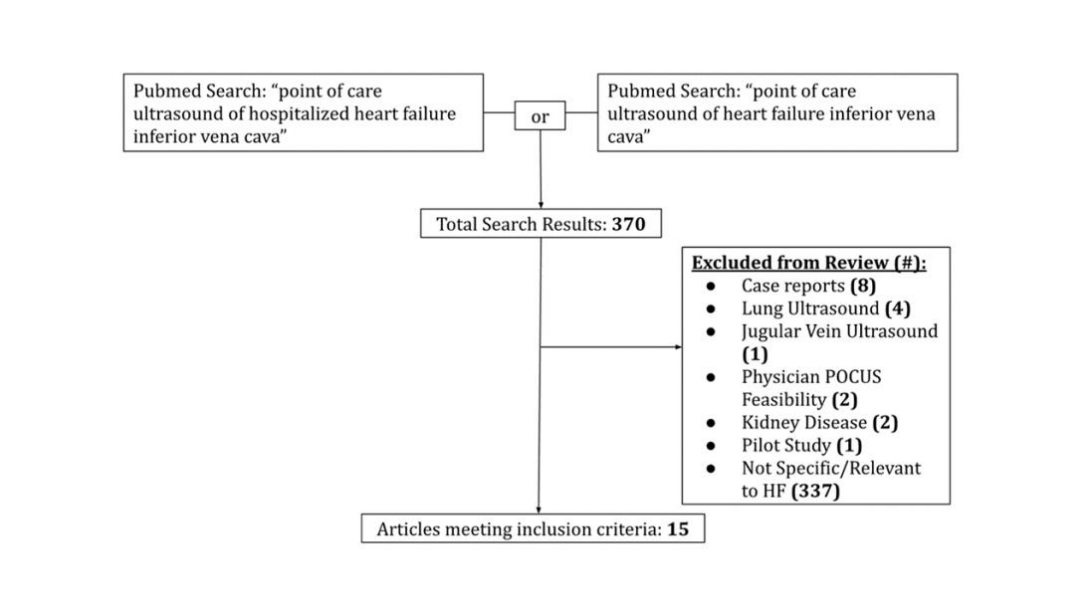
Search criteria figure. Results from targeted PubMed literature search using search terms “point of care ultrasound’ of “hospitalized heart failure inferior vena cava” or “point of care ultrasound” of “heart failure inferior vena cava”

The initial literature review with the combined databases resulted in a total of 370 journal articles. Of these, five studies were excluded because they did not assess IVC diameter; four studies focused only on lung ultrasound (US) and one study focused only on jugular vein ultrasound. Additionally, three studies were deemed outside the scope of the research objective (identifying the use of POCUS in managing HF), including one study that investigated physician training methods for POCUS and two that analyzed the use of POCUS for managing septic shock. Five case studies and one pilot study were excluded. The remaining 337 did not encompass all of the key terms included in the search query (i.e. not including either POCUS or HF in the body of the publication), thereby making their objectives and methodology out of the scope of this review. The remaining 15 studies all assessed POCUS as a diagnostic or maintenance tool for patients with HF and included IVC measurements as part of their criteria to some capacity (Figure 1).

### POCUS for the Diagnosis of Heart Failure

Of the 15 studies, five assessed the diagnostic capacity of POCUS for patients presenting with acute dyspnea (AD) in the emergency department (ED), compared with the clinical gold standard for HF diagnosis defined by abnormal chest x-ray, blood tests (such as proBNP), ECG, and clinical history (Table 1). Each of these studies calculated sensitivity, specificity, positive predictive value (PPV), negative predictive value (NPV), positive likelihood ratio (+LR), negative likelihood ratio (-LR), and accuracy. Additionally, these studies assessed the diagnostic capability of various combinations of measurement parameters determined using POCUS. These included IVC and IVC-CI, lung US measures such as B-line count (BLC) and B-zones (BBPC), left ventricular ejection fraction (LVEF), and abnormal or dilated cardiac chamber geometry. The presence of B-lines in the thorax – and, if in multiple thoracic zones, B-zones – indicates edema and left ventricular dysfunction i.e. reduced ejection fraction) 8. All studies showed similar measurement methods as well as diagnostic cut-off points.

Table 1 shows that the specificity of several measures and their combinations was increased relative totheir sensitivity. Miller et al. have demonstrated that as the cut-off for IVC-CI increases, the sensitivity of this measure increases while its specificity decreases. However, the particularly high +LR of 12.3 for an IVC-CI cut-off <10% suggests a potentially optimal cutoff for HF 9. Throughout all the studies, the highest seen sensitivity, specificity, PPV, and NPV were generally any combination of IVC-CI, LVEF, and lung US measures of BLC and bilateral B-pattern count (BBPC). Carlino et al. showed that any combination of the presence of bilateral ischemia (IS) (defined as ≥3 BLC), pleural effusion (PE) (defined as hypoechoic space between the two pleural walls), or a dilated left atria outperformed traditional diagnostic tools such as pro-BNP or chest x-ray in sensitivity, specificity, and accuracy 10.

Overall, the results show higher sensitivity and specificity in the diagnosis of HF with the use of POCUS compared to standard of care, specifically when combining cardiac, vascular, and lung US protocols and when utilizing conventional cut-off values for each parameter. The studies collectively showed that the specificity of POCUS was greater than its sensitivity, except for the caval-aortic ratio 9. The data indicates that the diagnostic power of POCUS is maximized upon the combination of IVC, LVEF, BLC, and BBPC, effectively utilizing both cardiac and lung radiography.

**Table 1:** Sensitivity, specificity, and other associated diagnosticstatistics  of various POCUS parameters used to diagnose patients with HF 9, 11, 12, 10, 13. Number of patients included in each study included in parentheses.

**Study** **(# of patients)**	**Diagnostic Parameter**	**Cut-point**	**Sensitivity (95%CI)**	**Specificity (95%CI)**	**Positive Predictive Value (PPV)**	**Negative Predictive Value (NPV)**	**+LR**	**-LR**	**Accuracy**
Miller et al (89)	IVC-CI	<10%	22%(11%-41%)	98%(89%-99%)	N/A	N/A	12.3	0.79	N/A
		<15%	37%(22%-55%)	96%(86%-99%)	N/A	N/A	10	0.64	N/A
		<25%	69%(51%-83%)	89%(77%-95%)	N/A	N/A	6.2	0.35	N/A
		<33%	80%(63%-91%)	81%(68%-90%)	N/A	N/A	4.2	0.25	N/A
		<40%	91%(76%-98%)	76%(62%-86%)	N/A	N/A	3.8	0.11	N/A
		<50%	94% (79%-99%)	59% (45%-72%)	N/A	N/A	2.3	0.09	N/A
	Caval-aortic Ratio	>0.4	99%(89%-100%)	2%(1%-10%)	N/A	N/A	1	0.51	N/A
		>0.6	94%(77%-99%)	13%(5%-26%)	N/A	N/A	1.1	0.46	N/A
		>0.8	84%(68%-95%)	52%(37%-66%)	N/A	N/A	1.8	0.32	N/A
		>1.0	57%(39%-74%)	81%(67%-90%	N/A	N/A	3	0.54	N/A
		>1.2	33%(18%-52%)	96%(86%-99%)	N/A	N/A	8.3	0.69	N/A
		>1.4	9%(2%-24%)	98%(90%-100%)	N/A	N/A	4.5	0.63	N/A
Farahamd et al. (120)	LVEF	< 45%	89.5%	92.1%	91.1%	90.6%	N/A	N/A	N/A
	IVC-CI	< 20%	35.1%	93.7%	83.3%	61.5%	N/A	N/A	N/A
	BLC	≥10 B-lines	73.7%	95.2%	93.3%	80.0%	N/A	N/A	N/A
	BBPC	≥ 2 zones	78.9%	93.7%	91.8%	83.1%	N/A	N/A	N/A
	LVEF and IVC-CI		33.3%	98.4%	95.0%	62.0%	N/A	N/A	N/A
	LVEF and BLC		68.4%	98.4%	97.55	77.5%	N/A	N/A	N/A
	LVEF and BBPC		73.7%	96.8%	95.5%	80.3%	N/A	N/A	N/A
	IVC-CI and BLC		33.3%	98.4%	95.0%	62.0%	N/A	N/A	N/A
	IVC-CI and BBPC		33.3%	98.4%	95.0%	62.0%	N/A	N/A	N/A
	BLC and BBPC		73.7%	95.2%	93.3%	80.0%	N/A	N/A	N/A
	LVEF and IVC-CI and BBPC		31.6%	98.4%	94.7%	61.4%	N/A	N/A	N/A
Carlino et al (102)	Chest x-ray	Chest x-ray		64.9% (47-79)	88.5% (77-95)	77.4% (59-90)	80.6% (69-89)	N/A	79.6%
	NT-pro-BNP	NT-pro-BNP		80% (63-91)	69.7% (51-84)	73.7% (57-86)	76.7% (57-89)	N/A	75%
	Bilateral IS and/or effusion	≥3 B-lines; hypoanechoic space between parietal and visceral pleura	100% (89-1000)	82% (70-90)	78% (64-88)	100% (91-100)	N/A	N/A	89%
	Dilated LA	Eyeball evaluation (anteroposterior diameter >4 cm)	92.3% (78-98)	77% (64-87)	72% (57-83)	94% (83-98)	N/A	N/A	83%
	LVEF	≤ 40%	59% (42-74)	90.2% (79-96)	79.3% (60-91)	77.5% (66-86)	N/A	N/A	78%
	Dilated LV	Eyeball evaluation	38.5% (24-55)	91.8% (81-97)	75% (51-90)	70% (59-80)	N/A	N/A	71%
	Abnormal LV geometry	Eyeball evaluation	84.6% (69-94)	80.3% (68-89)	73.3% (58-85)	89.1% (77-96)	N/A	N/A	82%
	IVC diameter; IVC-CI	Eyeball evaluation >2 cm; <50%	69.2% (52-83)	70.5% (57-81)	60% (44-74)	78.2% (65-88)	N/A	N/A	70%
	Bilateral IS and/or effusion & EF		59% (42-74)	100% (93-100)	100% (82-100)	79.2% (68-87)	N/A	N/A	84%
	Bilateral IS and/or effusion & dilated LA		94.9% (81-99)	93.4% (83-98)	90.2% (76-97)	96.6% (87-99)	N/A	N/A	94%
	Bilateral IS and/or effusion & either EF or dilated LA or both		100% (89-100)	93.4% (83-98)	90.7% (77-97)	100% (92-100)	N/A	N/A	96%
Zanobetti et al (2683)	LUS		88% (85.1-90.6)	96% (95-96.8)	85.8% (82.8-88.5)	96.6% (95.8-97.4)	21.73% (17.61-26.82)	0.12% (0.10-0.16)	N/A
IVC-CI = Inferior vena cava collapsibility index; Caval-aortic Ratio = Ratio of inferior vena caval diameter to aortic diameter; LVEF = left ventricular ejection fraction; BLC = B-line count; BBPC = Bilateral B-pattern count; NT-pro-BNP = N-terminal prohormone of brain natriuretic peptide; IS = ischemia; LA = left atria; LV = left ventricle; LUS = lung ultrasound; ECHO = echocardiogram; * = In addition, Zanobetti et. al reported an optimal concordance between ultrasound and ED diagnoses for HF of 0.8 < < 1, while significantly more sensitive (88% vs 77%; P < 0.001), and significantly faster in forming a diagnosis (24 ± 10 min vs 186 ± 72 min; P = 0.025). The difference in specificity between the two was insignificant (96% vs 98%; P < 0.001).									

### POCUS for the Management of Heart Failure

Ten studies evaluated the utilization of POCUS for the management of known HF patients (Table 2). Five of these studies compared the parameters measured with POCUS, primarily IVC measurement, with other variables using Spearman or Pearson tests. These reference variables included clinical assessment of volume status change (based on the resolution of peripheral edema) following administration or adjustment of diuretic dose as well as comparison to volume status determined from physical examination and reference echocardiography measurements. While most studies assessed the correlation between POCUS and the reference variables, Tchernodrinski et al. and Hacıalioğulları et al. assessed the change in IVC diameter relative to baseline at various time points following diuretic administration 14, 15.The reliability of POCUS as a tool compared to other methods of assessing volume status was tested by Nixon et al. and Dalen et al. Nixon et al. found a significant correlation between physical volume assessment and POCUS IVC_d_ measurements 13, and Dalen et al. found a significant correlation between POCUS and baseline echocardiography measurements of IVC_max_, IVC_min_, and determination of PE 16.

Tchernodrinski et al. and Hacıalioğulları et al. both assessed the ability of POCUS to identify a sonographic change in volume status of patients with HF following intravenous diuretic administration, with Tchernodrinski et al. identifying a significant change in IVC_max_ both 1-2 hours and 3-4 hours following the treatment 14. Hacıalioğulları et al. found similar results 3 hours following treatment not only for IVC_min_, IVC_max_, and IVC-CI, but also for the presence of B-lines in both right and left lung zones 15. During both the initial visit and follow-up, Gundersen et al. observed a correlation between POCUS-determined volume status (hypervolemic, hypovolemic, or euvolemic) and the alteration of diuretic dose, and a weak correlation between IVC_max_, IVC-CI, or the presence of PE and diuretic dosing (all p-values <0.05). Overall, these ultrasound parameters strongly correlated with nurse-assessed physical volume status and showed improvement with the administration of diuretics 17. The utilization of POCUS parameters in the management of HF produces more definite results than a physical examination by providing a reference quantitative measurement, which can be used to evaluate the effect of diuresis.

**Table 2:** Correlations between POCUS parameters and various comparison variables as well as evaluation of POCUS parameter change following diuretic administration 14, 18, 17, 16, 15. Number of patients included in each study included inparentheses .

**Study** **(# of patients)**	**Comparison Variable**	**Diagnostic** **Parameter**	**Time-point**	**Correlation Coefficient r/ Coefficient of Determination R^2^**	**P-Value **
Tchernodrinski et al. (70)	Administration of intravenous diuretic	IVC_max_	1-2 hrs following administration	N/A	<0.0001*
			3-4 hrs following administration		<0.0001*
Nixon et al.(150)	Physical Volume Assessment	IVC_d_	N/A	r=0.46	0.000
Gundersen et al.(62)	Diuretic Dose Adjustment	Volume Status based on POCUS	First visit	R^2^=0.375	<0.001
			Follow-up	R^2^=0.391	<0.001
		IVC-CI Score	First visit	R^2^=0.207	<0.001
			Follow-up	R^2^=0.062	<0.001
		IVC_max_ Score	First visit	R^2^=0.115	0.01
			Follow-up	R^2^=0.186	<0.001
		PE	First visit	R^2^=0.09	0.02
			Follow-up	R^2^=0.13	<0.01
	Volume Status	IVC_max_	N/A	r=0.67	<0.001
		PE		r=0.67	<0.001
Dalen et al. (62)	Reference Echocardiography Measurements (Reliability)	IVC_max_	N/A	r = 0.89	<0.001
		IVC_min_		r = 0.79	<0.001
		PE, left		r = 0.95	<0.001
		PE, right		r = 0.97	<0.001
		PE, both cavities		r = 0.96	<0.001
Hacıalioğulları et al. (80)	Treatment following initial HF diagnosis in the ED ☨	IVC_max_	3 hours following administration	N/A	<0.001
		IVC_min_			<0.001
		IVC-CI			<0.001
		B-lines (left and right zones)			<0.001
* Comparison was made between diagnostic parameter measurements at different time-points compared to baseline, rather than to a reference variable. Gundersen scoring (end-expiratory dimension scores 1, 2 and 3 refer to IVC_max_ <1.7 cm, 1.7–2.1 cm and >2.1 cm, respectively, and for the IVC-CI scores 1, 2 and 3 refer to ≥50%, 35-50% and <35%, respectively). IVC max = Maximum diameter of the inferior vena cava during end expiration; IVCd = Diameter of the inferior vena cava (unspecified if min or max or average); IVC min = Minimum diameter of the inferior vena cava during inhalation; PE = pulmonary effusion; IVC-CI = Inferior vena cava collapsibility index					

### Heart Failure Outcomes Prediction by POCUS

Akhabue et al., Khandwalla et al., Torres et al., Gustaffson et al., Goonewardena et al., and Hacıalioğulları et al. each evaluated the ability of POCUS to predict outcomes of patients with HF including readmission and composite endpoints of combined readmission or death (Table 3). The examined populations included adults who had been hospitalized for HF. The average age of these patients in each study was over 65, and the patient populations were well-diversified in both gender and race. Statistical methods for analysis included t-tests comparing outcomes as well as time-points (initial data collection vs. follow-up), ROC and Kaplan-Meier curves, and hazard, odds, and risk ratio calculations. Longitudinal studies analyzing survival and readmission likelihood assessed patients at two time points, comparing POCUS measurements at admission vs. discharge or at discharge vs. an outpatient follow-up visit within a year after discharge.

When comparing differences between outcome groups, Akhabue et al. found that differences in IVC-CI values were not significant between the no rehospitalization outcome vs. the rehospitalization and/or death outcome at discharge. However, the difference between the outcomes was significant at follow-up, indicating that POCUS is a better predictor of longevity at some time following the initial hospitalization event 19 (Table 3). Goonewardena et al. found IVC-CI was significant at discharge when applying a cut-off value for IVC-CI at <50% 20. Furthermore, Akhabue et al. found that IVC_max_ was not significant between readmission/death and no readmission groups both at discharge and follow-up. However, when utilizing a cut-off point of >2.0 cm, the IVC_max_ differences between readmission/death vs. no readmission outcomes were significant both between admission and discharge^20^ as well as between discharge and follow-up 19. This is supported by Khandwalla et al., who found a non-significant difference in IVC_max_ values between patient groups with or without a previous HF hospitalization when a cut-off value was not utilized, despite a significant difference between IVC_min_ and IVC_avg_ values between the two groups 21.

When considering differences between time points, Akhabue et al. found a significant difference in IVC_max_ at discharge and follow-up for patients who were not readmitted 19. Goonewardena et al. also demonstrated that utilizing an IVC cut-off value rendered a significant difference between readmission vs. no readmission outcomes for IVC-CI – in addition to pro-BNP – at discharge, while IVC_max_ was not significant both at admission and discharge. They suggested that the prognostic capacity of IVC measurements with POCUS is greater or at minimum equal to that of proBNP 20. Furthermore, Hacıalioğulları et al. found a significant difference in the IVC_max_ and IVC_min_ between HF patients who were discharged from the ED and those who were hospitalized during the initial scan taken in the ED, with mean IVC_min_ differences remaining significant following treatment administration during the final POCUS scan. POCUS of the right lung lobe also produced significant differences between these two outcomes during the initial and final scans. However, ejection fraction (EF) and BNP level differences remained non-significant 15.

Akhabue et al. observed a significant increase in the area under the ROC curve between discharge and follow-up for overall IVC_max_ values, the change between IVC_max_ values between discharge and follow-up, and IVC-CI values with a cut-point of <42% 19. The predictive power of IVC measurements is further supported by the findings of Goonewardena et al., who noted large areas under ROC curve for an IVC_max_ > 2.0 cm, IVC-CI<38%, and proBNP > 2,327. Goonewardena et al. also found an odds ratio of 6.1 for logBNP levels > 3.367 and 10.3 for an IVC_max_ > 2.0 cm 20.

When evaluating survival, Torres et al. found a significant difference in patients below the cut-off value of IVC_max_ ≥2.3 cm vs. patients above the cut-off, as well as for patients both above the IVC_max_ cut-off and below the mean arterial pressure (MAP) cut-off of < 93.3 mmHg independent of echo-based LVEF 22. Khandwalla et al. found an increased risk ratio for patients with a mean IVC between 2.0 cm and 2.5 cm, and an additional 14% and 38% increased risk seen with IVC diameters 0.2 cm and 0.5 cm above 2.5 cm respectively in the risk of HF hospitalization (p < 0.05) 21. However, Gustaffson et al. did not observe a significant difference in patients above or below an IVC_max_ cut-off of >1.8 cm, but found a significantly reduced survival for patients determined to have either comet tail artifacts (CTA) and/or PE 23. Akhabue et al. found a significantly greater hazard ratio of 6.8 for patients with an IVC-CI <42% 19.

Four studies – Khandwalla et al., Torres et al., Gustafsson et al., and Goonewardena et al. – determined the correlation coefficient between POCUS measurements and proBNP/logBNP levels, NYHA class, atrial fibrillation, and chronic ischemic heart disease. The results used were those adjusted for other variables such as mean weight, age, and atrial fibrillation.

These studies determined significant, but weak, correlations between IVC_d_ and proBNP/logBNP levels, as well as between CTA or PE and proBNP/logBNP levels. Additionally, Gustaffson et al. found a similar correlation between CTA or PE and New York Heart Association (NYHA) class 23, while Torres et al. found a weak positive correlation between IVC_max_ and atrial fibrillation as well as a weak negative correlation between chronic ischemic cardiac disease 22.

**Table 3:** Collection of statistical measures assessing the ability of POCUS in the assessment of hospitalization or combined hospitalization and mortality 15, 19, 21, 22, 23, 20. Number of patients included in each study included in parentheses.

Study (# of Patients)	Outcome	Diagnostic Parameter	Cut-Point	Time-Point	P-value Between Outcomes (<0.05)	P-value Between Time Points (<0.05)	Area Under ROC or Kaplan-Meier Curve/ p-value	Hazard/ Risk Ratio/Odds ratio ( 95%CI)
Akhabue et al. (49)	Readmission or death vs. no readmission	IVC_max_	>2.1 cm	Discharge	NS	**Readmission/death:** NS **No readmission: **0.038	**With Cut-point: **0.57 **Without Cut-point:** 0.58	N/A
				Follow-up	NS		**With Cut-point: **0.60 **Without Cut-point: **0.66 **Change between Discharge and Follow-up: **0.65	N/A
		IVC-CI	<50%	Discharge	NS	**Readmission/death:** NS **No readmission: **NS	With Cut-point: 0.62 Without Cut-point: 0.69	N/A
				Follow-up	0.040		With Cut-point: 0.60 Without Cut-point: 0.66 Change between Admission and Follow-up: 0.59	N/A
			<42%	Follow-up	N/A	N/A	With Cut-point: 0.73	6.8 (2.4–19.0)
		IVC_max_ + IVC-Ci	N/A	Discharge	N/A	N/A	**With Cut-point:** N/A **Without Cut-point: **0.66	N/A
				Follow-up			**With Cut-point**: N/A **Without Cut-point: **0.72	N/A
Khandwalla et al. (355)	1+ hospitalization vs. no hospitalization	Mean IVC_d_	<2.0 cm	N/A	<0.01	N/A	N/A	1.0
			2.0 - <2.5 cm					1.79 (1.27-2.52)
			≥2.5 cm					2.39 (1.55-3.67)
			Per 0.2 cm increase					1.14 (1.06-1.21)
			Per 0.5 cm increase					1.38 (1.16-1.62)
			Per 1.0 cm increase					1.89 (1.36-2.64)
		IVC_max_	Per 0.2 cm increase		NS			1.02 (0.96-1.10)
			Per 0.5 cm increase					1.06 (0.90-1.26)
			Per 1.0 cm increase					1.13 (0.81-1.58)
		IVC_min_	Per 0.2 cm increase		<0.01			1.13 (1.07-1.19)
			Per 0.5 cm increase					1.36 (1.19-1.54)
			Per 1.0 cm increase					1.84 (1.42-2.38)
Torres et al. (123)	Mortality vs. no mortality	IVC_max_	≥ 2.3 cm	N/A	N/A	N/A	P-value: 0.0070	**Univariate:** 1.06 (1.02-1.11) **Multivariate: **1.06 (1.01-1.10)
			≥ 2.3 cm and MAP < 93.3 mmHg				P-value: 0.0003	
		LVEF	N/A				N/A	**Univariate:** 0.98 (0.96-1.00) **Multivariate: **0.98 (0.96-1.00
Gustafsson et al. (104)	Hospitalization or death vs. no hospitalization or death	IVC_max_	>1.8 cm	N/A	N/A	N/A	P-value: NS	N/A

		CTA	>3 comet tails				P-value: 0.003	**Model 1:** 3.5 (1.5-7.9) **Model 2:** 2.9 (1.3–6.6)	
		PE	Present vs. absent				P-value: 0.009	**Model 1:** 3.9 (1.4–10.8) **Model 2: **1.9 (0.6–6.2)	
		CTA or PE					P-value: 0.002	**Model 1: **3.7 (1.6–8.5) **Model 2: **4.9 (1.2–20.1)	
Goonewardena et al. (75)	Readmission vs. no Readmission	BNP	2,327	Admission	N/A	N/A	0.69	N/A	
				Discharge	N/A				
		logBNP	3.367	Admission	0.28	N/A	N/A	Odds: 6.1	
				Discharge	0.04				
		IVC_max_	>2.0 cm	Admission	0.02	N/A	0.78	Odds: 10.3	
				Discharge	0.001				
		IVC_min_	N/A	Admission	0.03	N/A	N/A	N/A	
				Discharge	<0.001				
		IVC-CI	<50%	Admission	0.10	N/A	0.74	N/A	
				Discharge	0.002				
Hacıalioğulları et al. (80)	Discharged from ED vs. Admitted to hospital	BNP	N/A	N/A	NS	N/A	N/A	N/A	

		EF %		N/A	NS				

		IVC_max_		Initial Scan	0.042				
				Final Scan	NS				
		IVC_min_		Initial Scan	0.014				
				Final Scan	0.025				
		IVC-CI		Initial Scan	NS				
				Final Scan	NS				
		Right Lung Zones		Initial Scan	**Zone 1: **0.016 **Zone 2:** 0.01 **Zone 3:** NS				
				Final Scan	**Zone 1: **0.001 **Zone 2:** 0.012 **Zone 3:** 0.006				
		Left Lung Zones		Initial Scan	**Zone 1: **0.028 **Zone 2:** NS **Zone 3:** NS				
				Final Scan	**Zone 1: **0.004 **Zone 2:** NS **Zone 3:** NS				
		PE, Right and Left		Initial Scan	NS				
				Final Scan	NS				
IVC max = Maximum diameter of the inferior vena cava during end expiration; IVC-CI = Inferior vena cava collapsibility index; IVC min = Minimum diameter of the inferior vena cava during inhalation; Mean IVCd = Average diameter of the inferior vena cava during the respiration cycle; CTA = comet tail artifact; PE = pulmonary effusion; BNP = Brain natriuretic peptide; logBNP = logarithm of brain natriuretic peptide value; EF % = ejection fraction in percentage									

## Discussion

**Figure 2:**
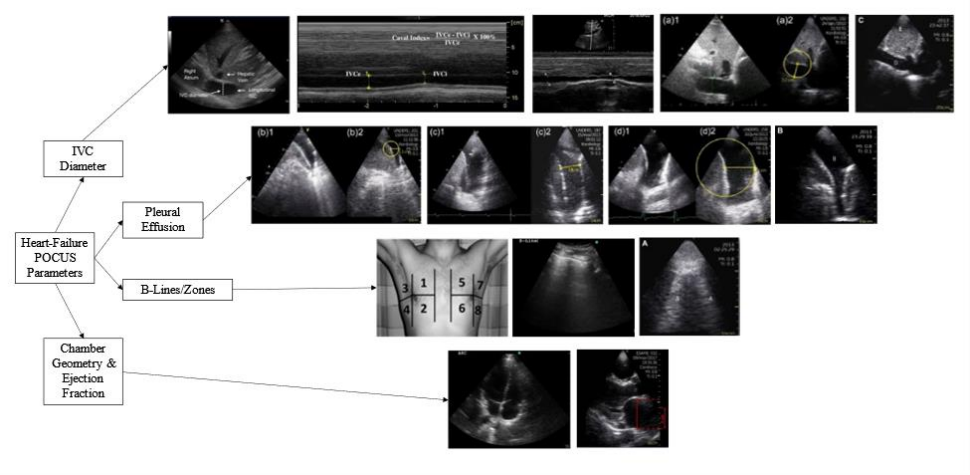
Collection of Images Presented Using Selected POCUS Measurement Parameters. Top to Bottom: IVC Diameter, PE, B-Lines/Zones, Chamber Geometry & Ejection Fraction. Left to Right Row 1 Miller et al., localization of IVC and IVC-CI calculation in M-Mode;Farahmand et al., IVC diameter measurement in M-mode; Dalen et al , end-expiratory IVC diameter (IVC_max_); Gustaffson et al., IVC diameter measurement. Left to Right Row 2 Gundersen et al., PE in the cost diaphragmatic angle, lower lobes bulging into the effusion, and a significant excess of PE; Gustaffson et al., bilateral PE in the infrascapular region. Left to Right Row 3 Anderson et al, demarcation of the 8 thoracic zones considered in B-zone scoring; Farahmand et al., One B-line in the superior anterior right zone; Gustaffson et al., multiple B-lines/comet tail artifacts. Left to Right Row 4 Farahmand et al., 4-chamber view used to estimate ejection fraction; Carlino et al., Anteroposterior diameter of dilated left atrium in the parasternal long-axis view 9, 11, 10, 17, 23.

The collection of evidence from this review suggests that IVC diameter assessment with POCUS is a useful diagnostic tool in terms of both sensitivity and specificity compared to historical methods utilized for patients presenting with AD in the ED. IVC measurement and IVC-CI calculations are particularly effective in determining diagnosis when complemented with lung ultrasound examining B-lines and the presence of PE. Despite loosely correlating with other HF markers such as physical examination assessment of volume status and pro-BNP, POCUS IVC and IVC-CI demonstrate effects of diuretic administration, making them a quick, non-invasive, and relatively easy method to assess treatment effect for both hospitalized and outpatient HF patients (Figure 2).

Furthermore, IVC diameter measurements, particularly when coupled with a lung ultrasound scan, may serve as a prognostic tool in predicting readmission or mortality in hospitalized HF patients. The results from Table 3 show that an IVC > 2.0 cm is generally associated with significantly increased risk; this is corroborated by Akhabue et al. who found the same for a calculated IVC-CI < 42%. Studies evaluating the predictive ability of POCUS also found that the presence of significant interstitial lung fluid (defined as >3 B-lines) and/or PE in the thoracic cavity reflects worse patient prognosis. As POCUS becomes fully integrated as a standard of care for hospitalized HF patients, these markers can signal the need for more intensive patient monitoring and treatment plans. While Goonewardena et al. show that logBNP measures have similar predictive capacity for readmission as POCUS, using POCUS is potentially more feasible in more dynamic clinical environments. POCUS is non-invasive and provides instant visual results that can be captured, shared, and compared between users and serially over time.

POCUS as both a diagnostic and management tool can be used to image anatomic changes over time and reflect changes in physiology. Pathophysiologic changes associated with the onset of HF and treatment of HF can be observed as quantifiable changes in anatomy as seen with POCUS. An increase in measured IVC diameter correlates with an increase in CVP 24. Therapies that reduce or normalize CVP are central to the clinical management of HF 25. An increase in CVP is caused by reduced output into arterial circulation and a backing up of blood in venous circulation, as well as fluid retention due to reduced renal function 26. The presence of B-lines and PE demonstrate physiologic responses to volume overload associated with HF that can result in lung congestion and reduced respiratory capacity 27. While POCUS has the capacity to acquire this information at the bedside, physical examinations provide more limited assessments of volume status and cannot quantitatively assess volume changes. This limitation is often insufficient to reliably manage acute and chronic HF 28.

The simplicity, accuracy, and reproducibility of POCUS is ever-increasing along with advancements in technology and imaging systems, such as automated tissue differentiation tools that can enhance image resolution and image assessment and increase clinical accuracy of bedside POCUS diagnoses. As the POCUS technology and user experience grows, ongoing investigations and use-cases for POCUS in varying clinical settings and scenarios will expand. The expansion of scientific study with POCUS as a dynamic imaging tool will intensify with ongoing improvements in technology and the decreased cost of devices and software. Standardized workflows and image acquisition and measurements can further the study and demonstrate pathways for differential outcome assessment in HF management.

**Figure 3:**
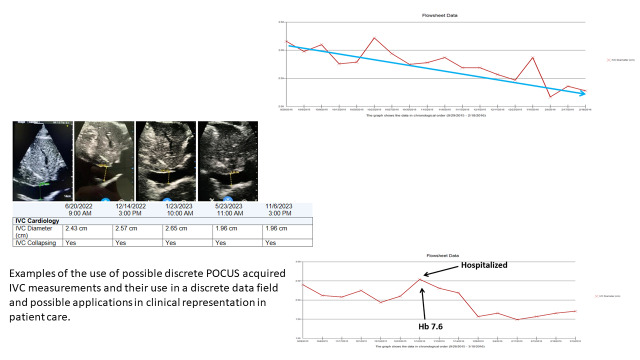
Examples of the use of point of care ultrasound (POCUS) acquired inferior vena cava (IVC) measurements and their use in a discrete data field within the electronic health record and for representation of these data for patient care applications.

Including a review of the current research that demonstrates the merits of POCUS-use in the management of HF patients highlights opportunities for future study. POCUS and its regular use warrants exploration for the development of a systematic methodology and simplified POCUS measurement set to study and standardize for scaling up the clinical use of POCUS both for HF management and other clinical conditions. We offer a possible option to standardize a specific assessment and measurement of the IVC that can be shared and compared as a discrete data field with an accompanying image in clinical care notes and the shared patient medical record. As with a standard vital sign, this IVC assessment can have a generalized range and a patient specific range that represents physiologic and anatomic changes (Figure 3).

Additionally, with the inevitable improvement in POCUS image acquisition, image quality, user ubiquity, and user comfort, there is opportunity for future studies to explore POCUS strategies for chronic disease management and tighter control and maintenance of euvolemia in HF patients. This review of the use of POCUS in hospitalized HF patients may offer insights for future investigators to generate research hypotheses for future study. 

## Conclusion

The review suggests that POCUS, and particularly the measurement of the IVC, can serve as a useful tool to diagnose HF   in patients presenting with AD in the ED, to monitor the efficacy of diuretic administration and predict dose adjustments, and to evaluate the prognosis of patients hospitalized with HF. The ability of POCUS in both diagnostic and treatment settings is likely optimized when combining IVC measurements with lung US parameters. 

## Disclosure statement

The authors report no relevant disclosures or conflicts of interests related to this work.

## Supplementary Material

 Appendix A and Appendix B Appendix A. Boolean Search Strategy; Appendix B. Table 4. Correlations between POCUS parameters and other markers of HF assessed and heart disease.
