# Supplementary material for: Point of Care Ultrasound for Diagnosis and Management in Heart Failure: A Targeted Literature Review
Source: POCUS J. 2024 Apr 22;9(1):117–30. doi: 10.24908/pocus.v9i1.16795 (PMC11044942; doi:10.24908/pocus.v9i1.16795)
Supplement: Appendix A and Appendix B [file pocusj-09-16795-s001.pdf]

Appendix A: Boolean Search Strategy.

1. Observational study (a)
2. Prospective cohort study (a)
3. Retrospective cohort study (a)
4. Randomized controlled trial (a)
5. Comparative study a)
6. 1 or 2 or 3 or 4 or 5
7. Point of care ultrasound (POCUS) (b)
8. Heart Failure (HF) (b)
9. Inferior Vena Cava (IVC) (b)
10. Hospitalized (b)
11. 7 and 8 and 9
12. 7 and 8 and 9 and 10
13. 11 or 12
14. Non-English (c)
15. Child studies (c)
16. Case Studies (c)
17. 14 and 15 and 16
18. 13 not 17

a = types of studies; b = search terms; c = exclusion criteria

Appendix B: Table 4. Correlations between POCUS parameters and other markers of HF assessed and heart disease [20-23].

Study (# of Patients)		logBNP/pro-BNP		NYHA Class		Atrial Fibrillation		Chronic Ischemic heart Disease	
		Correlation coefficient r	p-value	Correlation coefficient r	p-value	Correlation coefficient r	p-value	Correlation coefficient r	p-value
Goonewardena et al.	IVC_{max}	r = 0.23	N/A	N/A	N/A	N/A	N/A	N/A	N/A
	IVC-CI	r = -0.12	N/A						
Torres et al.	IVC_{max}	N/A	N/A	N/A	N/A	r = 0.232	0.0092	r = -0.200	0.0244
Khandwalla et al.	Mean IVC_d	r = 0.11	< 0.01	N/A	N/A	N/A	N/A	N/A	N/A
Gustafson et al.	CTA	r = 0.28	< 0.01	r = 0.21	< 0.05	N/A	N/A	N/A	N/A
	PE	r = 0.37	< 0.001	r = 0.20	< 0.05				
	IVC_{max}	r = 0.26	< 0.05	r = 0.05	NS				
	IVC_{max} > 2.4 cm	r = 0.24	< 0.05	r = −0.03	NS				
